# Can the Rorschach be Administered Remotely? A Review of Options and a Pilot Study Using a Newly Developed R-PAS App

**DOI:** 10.1007/s12207-022-09447-z

**Published:** 2022-03-16

**Authors:** Francesca Ales, Gregory J. Meyer, Joni L. Mihura, Andrea Corgiat Loia, Sara Pasqualini, Alessandro Zennaro, Luciano Giromini

**Affiliations:** 1 Department of Psychology, University of Turin, Via Verdi 10, 10123 Turin, TO Italy; 2 Department of Psychology, University of Toledo, Toledo, OH USA

**Keywords:** Rorschach, R-PAS, Tele-health, Personality, Assessment

## Abstract

The ongoing COVID-19 pandemic has required psychologists to adopt measures like physical distancing and mask wearing, though other safety procedures such as travel restrictions or prohibitions on in-person practice and research have fostered the use of tele-health tools. In this article, we review options for using the Rorschach task via videoconference and provide preliminary data from using a new electronic app for remote R-PAS administration to determine whether the remote administration in an electronic form yields different information than in-person administration with the cards in hand. As a pilot study, our focus is on the “first factor” of all Rorschach scores, i.e., complexity. Data were collected from 60 adult Italian community volunteers, and statistical analyses evaluated the extent to which the average complexity score significantly departed from R-PAS normative expectations (SS = 100), accompanied by Bayesian likelihoods for supporting the null hypothesis. Results suggest that the general level of complexity shown by the test-takers when administered the Rorschach remotely with the new R-PAS app closely resembles that previously observed using “standard” in-person procedures. Tentative analyses of other R-PAS scores suggested normative departures that could be due to the effects of the app, testing at home, or responses to the pandemic. We offer recommendations for future research and discuss practical implications.

## Introduction

The spread of COVID-19 has dramatically changed the landscape of mental health services around the world, strongly affecting the ways in which psychological assessment used to be conducted. Over the past 2 years, preventive and corrective measures to control the COVID-19 outbreak have caused difficulties in delivering basic mental health care services. In May 2020, the Society for Personality Assessment conducted a survey, the results of which were quite discouraging: 26% of practitioners conducted assessment procedures virtually, via videoconferencing, but 52% paused their psychological evaluations, waiting until in-person activities could be resumed. Even with vaccine dissemination, the scenario of mental health services seems to have changed radically and definitively. Thus, it is necessary to adapt psychological services to the new tele-assessment context. In fact, psychological assessment has had difficulty adapting quickly to the pandemic context, especially compared to psychotherapeutic treatment practice that has moved quite easily to the online mode, thanks to studies that empirically supported it (Batastini et al., 2016; Bolton & Dorstyn, 2015; Reese et al., 2015; Varker et al., 2019). Therefore, in the last 2 years, increasing attention has been paid to the development of tele-health^1^ practices (i.e., delivery of health care services via remote technologies).

It should be specified that although tele-health research has been more prolific recently and the health emergency has certainly increased interest in it, researchers have been studying these procedures over the past 20 years (Barnett et al., 2018; Spivak et al., 2020; Wilson et al., 2017). Initially, the appeal of tele-health was linked to the desire to improve equity and access conditions for those who could not easily travel (e.g., the elderly, people living in rural areas). In fact, prior to the ongoing pandemic, best practice guidelines for virtual psychological assessment had been published (Joint Task Force, 2013; Luxton et al., 2014), and some psychological measures had been tested with equivalence analyses comparing in-person and remote assessment.

Overall, research on tele-assessment has produced encouraging results regarding the reliability, validity, and utility of psychological data collected remotely. For example, several studies have showed that structured interviews conducted remotely are equivalent to traditional interviews conducted in-person, both in clinical and forensic settings (Garb, 2007; Grady et al., 2011; Hyler et al., 2005; Lexcen et al., 2006; Luxton et al., 2014; Manguno-Mire et al., 2007; Schopp et al., 2000; Shore et al., 2007; Singh et al., 2007). This is likely due to the fact that the success of a clinical interview is largely related to the degree of therapeutic alliance (COVID-19 Task Force to Support Personality Assessment, 2020), which appears to be undiminished in tele-health practices (Germain et al., 2010; Morgan et al., 2008; Simpson, 2001). Similarly, fairly strong evidence has demonstrated equivalence between self-report measures administered remotely and in-person (Garb, 2007; Giromini et al., 2021; Luxton et al., 2014), although it is necessary to ensure that test integrity is maintained (Corey & Ben-Porath, 2020). For example, the Millon Clinical Multiaxial Inventory 4th Edition (MCMI-IV; Millon et al., 2015) has been found to have good equivalency when administered electronically (Finger & Ones, 1999). Finally, several studies have focused on equivalency analyses of neuro-psychological tests (Cullum et al., 2006, 2014; Galusha-Glasscock et al., 2016; Grosch et al., 2015; Harrell et al., 2014; Loh et al., 2007; Temple et al., 2010; Tukstra et al., 2012). In a recent meta-analysis, Brearly et al. (2017) observed that videoconferencing administration does not result in significantly different outcomes compared to in-person administration.

According to the SPA survey (2020), the main pitfall many clinicians reported was the remote administration of some psychological measures, especially the performance-based ones, e.g., cognitive tests. A few attempts were made to assess possible differences between online *vs* traditional in-person administration for cognitive tests, but most have focused on specific tasks, i.e., WAIS-IV subtests (Brearly et al., 2017; Temple et al., 2010). In addition, most of these studies were conducted in highly controlled environments in which, for example, a facilitator was present to assist with test administration. Nonetheless, these studies represent the first efforts to demonstrate equivalence between tests administered in-person and tests administered remotely. Therefore, to date, one might say that there is more empirical support for online assessment than for in-person assessment with social distancing measures (e.g., masks, wider distance between assessor and client), on which no research has been conducted yet, and although research on tele-assessment is still young, it offers some empirical bases to build on.

Finally, it is worth mentioning the current status of tele-assessment in forensic contexts. The field lacks robust guidelines for online forensic assessment. Drogin (2020) pointed out that the courts have not yet become part of the debate about the use of tele-assessment in forensic evaluations, but he anticipated that this will happen soon. Thus, best practices and guidelines should be developed as soon as possible so that all parties involved (e.g., forensic psychologists, judges, attorneys) are able to handle these new tele-assessment practices.

### Rorschach and Tele-Assessment

Assessment instruments adapted to online administration from in-person administration inevitably introduce a risk of error as the instrument was validated under different assumptions, testing environments, and administration standards, all of which could affect the psychometric accuracy of the test (Kline, 2015). In particular, performance-based tests, which often use visual, tangible, or interactive stimuli instead of verbal items, have greater difficulty adapting to the online setting. To date, the only recommendations on how to conduct remote administration when dealing with performance-based personality assessment measures relate to the Rorschach task (Meyer et al., 2020). Meyer and colleagues’ guidelines (2020) refer specifically to the Rorschach, but can actually be applied to other performance-based tests in which the examinee interacts with visual stimuli.

Meyer et al. (2020) noted that online administration of the Rorschach test might generate a number of challenges, which can be easily guessed: the assessor cannot simply hold the cards and show them to the respondent via video camera, as the size of the stimuli and the respondent’s ability to rotate the stimuli are crucial features for its standardization. Sending the cards to the examinee can also pose some challenges. This option would open up the risk of possible violations of test security, the cards might not be returned to the clinician who would incur a financial loss, and the examinee would be wholly responsible for the entire test administration process. Other options for remote administration were proposed, such as the presence of an onsite facilitator (e.g., a professional, quasi-professional, a family member, or other cohabitant) who can receive the material and prepare the setting. More information about the potential issues with remote administration of the Rorschach and the solutions proposed by Meyer et al. (2020) is discussed below (see the paragraph “R-PAS at the time of COVID-19”).

### Rorschach Performance Assessment System (R-PAS)

Despite a long-standing debate on its validity and usefulness (e.g., Cronbach, 1949; Jensen, 1965; Lilienfeld et al., 2000; Meyer & Archer, 2001; Mihura et al., 2013, 2015; Society for Personality Assessment, 2005; Viglione, 1999; Viglione et al., 2022; Wood et al., 2015), the Rorschach inkblot task remains one of the most widely used (Wright et al., 2017), taught (Belter & Piotrowski, 2001; Childs & Eyde, 2002; Mihura et al., 2017), and researched^2^ assessment instruments. According to Meyer et al. (2011), one of the reasons for its popularity is that it offers a unique range of information about one’s personality features and processing style, giving the assessor the possibility to observe *performance*-related characteristics that are *typical*, rather than *maximum*.^3^

It should be underscored that from a psychometric standpoint, the claim that the Rorschach “is invalid” has been refuted by the most recent and extensive reviews and meta-analyses on this topic (Bornstein, 1999; Diener et al., 2011; Graceffo et al., 2014; Jørgensen, 2000; Meyer & Archer, 2001; Mihura et al., 2013, 2015; Viglione et al., 2022). In particular, Mihura et al.’s (2013) meta-analytic examination of all interpreted variables included in the popular comprehensive system (CS; Exner, 2003) led to the conclusion that 26 of them demonstrated *excellent* (*r* ≥ 0.33, *p <* .001, *FSN* > 50) or *good* (*r* ≥ .21, *p* < .05, FSN ≥ 10) validity, when using externally assessed criteria to evaluate them rather than self-report.^4^

Moreover, during the past two decades, several Rorschach variables (e.g., human movement, complexity, Vista) have received extensive empirical validation using (a) neuroscientific techniques such as EEG (Ando’ et al., 2018; Giromini et al., 2010; Pineda et al., 2011; Porcelli et al., 2013), fMRI (Asari et al., 2010; Giromini et al., 2017, 2019a, 2019b; Vitolo et al., 2020), and rTMS (Ando’ et al., 2015, 2018), or (b) psychophysiological research methods involving the measurement of electrodermal activity (Giromini et al., 2016), eye movements (Ales et al., 2019; Minassian et al., 2005, Perry et al., 1995), 5-hydroxyindoleacetic acid (5-HIAA) (Lundbäck et al., 2006), and other physiological criteria (Meaney, 2011; Meyer et al., 2018).

Therefore, selected scores from the Rorschach task may now be considered useful tools in the field of psychological injury and law (Erard, 2012; Erard et al., 2014; Erard, & Viglione, 2014; Viglione et al., 2022). Especially in forensic settings, it is crucial to ensure that a test used is empirically supported and has good psychometric properties, so that forensic examiners can draw valid and accurate conclusions from methods that meet current requirements for admissibility in court (Erard et al., 2014; Meyer & Eblin, 2012; Viglione et al., 2022). These selected scores are included in the Rorschach Performance Assessment System (R-PAS; Meyer et al., 2011, 2014; Meyer, & Eblin, 2012), which is the most updated method for administration, scoring, and interpretation and was designed as a replacement for the CS.

### R-PAS in Psycho-Legal Contexts

The use of the Rorschach in forensic practice is well-documented in the literature (Erard, 2012; Khadivi & Barton Evans, 2012; Meyer & Eblin, 2012; Mihura, 2012), and its use in the evaluation of psychological injury cases in particular is known to offer many advantages (Gacono & Evans, 2008). One of the strengths of using the Rorschach in forensic settings is that it is a viable alternative to self-reports. Most of the interviews (e.g., structured clinical interview for DSM-5: SCID-5; First et al., 2015) and personality inventories (e.g., the Minnesota Multiphasic Personality Inventory-3: MMPI-3; Ben-Porath & Tellegen, 2020; Personality Assessment Inventory: PAI; Morey, 1991) rely on the self-awareness of the defendant, on their ability to reflect on their own experiences, and to communicate their own personality characteristics honestly. The Rorschach, instead of asking how the respondent sees themselves, allows the assessor to observe how they see, communicate about, and interact with the inkblots, and thus does not rely on their ability of self-observation and self-awareness.

In this regard, it is worth specifying that the forensic context itself might be a stressful factor for the defendant. As much as one is willing to be honest in presenting their own memories and symptoms, it might be difficult not to emphasize their own discomfort and underestimate their own faults—if any. The Rorschach, as a free-response performance test, assesses what the person *does*, not what the person *says they do* (Meyer et al., 2011). It is not as clear to defendants how to deliberately appear cognitively disturbed or emotionally distressed during the Rorschach task. The opposite is also true: some individuals may have extremely resistant defensive barriers that cannot be overcome with self-report measures, but that can be scratched by methods such as the Rorschach, which are capable of showing psychopathology that is not overtly manifest (Ganellen, 2008; Ganellen et al., 1996; Grossman et al., 2002). Furthermore, self-reports can be redundant due to their shared mono-method variance (Meyer, 1999; Meyer et al., 2000). The Rorschach instead, confirming or disconfirming the results from self-reports, provides incremental validity (Weiner, 1999), and protects against the possibility that the defendant intentionally exaggerates or downplays certain aspects of their personality.

Another advantage of the Rorschach in forensic settings is that it is an implicit measure of personality traits and behavioral tendencies (Bornstein, 2002; McClelland et al., 1989; Shedler et al., 1993). Traditionally, implicit measures have been found to be useful in predicting how a person might behave in daily life, outside of structured settings such as psycho-legal evaluations, in which one might be inclined to meet particular expectations (Bornstein, 2002; Finn, 2011; McGrath, 2008). Finally, the Rorschach offers an idiographically rich and multifaceted representation of the defendant’s personality, which would be difficult to obtain through self-reports only (Erard, 2012).

In light of these strengths, the Rorschach is one of the ten most frequently used tools in forensic assessments of various kinds (Ackerman & Ackerman, 1997; Archer et al., 2006; Boccaccini & Brodksy, 1999; Borum & Grisso, 1995; Neal & Grisso, 2014; Quinnell & Bow, 2001) and ranks second in child custody evaluations.^5^ Furthermore, it is used in nearly a third of assessments to investigate criminal responsibility and competency to stand trial (Borum & Grisso, 1995), and nearly a third of forensic psychologists currently use it in their daily practice (Archer et al., 2006). Moreover, it is important to underline that even following the numerous controversies over the Rorschach, almost all the federal and state courts have considered it a sufficient tool for expert testimony (Meloy et al., 1987; Meloy, 2008; Viglione et al., 2022; Weiner et al., 1996).

Some might argue that R-PAS is too young a method to be accepted in legal processes. However, there are many reasons why R-PAS is commonly accepted in psycho-legal contexts. R-PAS has more than 9000 registered account holders, with more than 600 of them approved for teaching purposes. Account holders reside in every US state and 56 other countries. The R-PAS manual (Meyer et al., 2011) is in its tenth printing, and it is accompanied by a 19-chapter book illustrating case interpretation, including in forensic practice (Mihura & Meyer, 2018). The manual has been translated into four languages (Italian, Japanese, Portuguese, and Spanish) with five others in progress (Czech, Complex Chinese, Hungarian, Korean, and Thai). The online scoring program and resource center are available in 14 languages, and R-PAS has contracted local distributors and account brokers in seven countries. Finally, R-PAS has offered more than 180 official training workshops throughout the USA and in 17 other countries. Thus, with this scope of active use, it appears that R-PAS meets a standard of general acceptance with respect to its clinical use. This is in addition to the many ongoing studies and published research supporting R-PAS and the continuous increase in citations of the method by clinical and forensic textbooks (e.g., Ackerman & Kane, 2011; Archer & Smith, 2014).

Lastly, US federal law provides the use of the Daubert standard as a rule of evidence for the admissibility of expert witness testimony. What has become known as the Daubert trilogy (i.e., the three US Supreme Court cases that articulated the Daubert standard: Daubert, 1993; General Electric v. Joiner, 1997; Kumho Tire, 1999) stipulates seven non-exclusive and non-mandatory criteria that can be applied in a rather flexible manner in assessing the scientific reliability of expert testimony. In light of the seven Daubert criteria:

1. R-PAS is a testable technique (1st criterion). It is an evidence-based method whose results can be tested by means of countless techniques (e.g., convergent and discriminant correlations with behavioral measures).
2. R-PAS has been developed and tested (Mihura et al., 2013; also, see the Technical Manual section in Meyer et al., 2011) through extensive and supportive empirical testing of validity and reliability (2nd criterion).
3. R-PAS has been and still is subject of peer review (3rd criterion). The Rorschach is the second most studied assessment tool after the MMPI, and R-PAS specifically is based on peer-reviewed research (Meyer et al., 2012).
4. R-PAS variables have been tested to estimate a potential error rate (4th criterion). Interrater reliability is excellent on average (Schneider et al., 2020), indicating generally minimal error when classifying Rorschach responses, and Mihura et al. (2013) report an overall validity effect size (*r* = .27) within the typical range of variable-to-criterion error rates for psychological testing (Hemphill, 2003).
5. R-PAS uses standardized rules of administration (e.g., R-optimized administration) and, in order to maximize validity and reliability, has eliminated all variables that are not scientifically supported (5th criterion).
6. R-PAS is a relatively new method, but its popularity and use in training and assessment contexts are extensive (6th criterion). The comprehensive system (Exner, 1974), R-PAS’ predecessor, is generally accepted in nearly all courts; therefore, R-PAS, which originated from the CS, but improves its scientific foundations, can aptly anticipate receiving the acceptance that the CS has already benefitted (Erard et al., 2014). Additionally, Daubert standards are open to innovations with respect to old methods.
7. Chapter 10 of the R-PAS Manual (Meyer et al., 2011) offers specific guidelines on what inferences can be made using the test. Thus, with respect to whether the expert’s conclusions reasonably follow from applying the technique (7th criterion), it is partly up to the expert in the particular legal case to assess whether the use of the R-PAS can be helpful to the judge.

### R-PAS at the Time of COVID-19

Like all assessment measures involving the interaction between an assessor and a respondent, the ongoing COVID-19 pandemic has posed challenges to the Rorschach’s use in applied settings, due to the need for physical distancing and other safety procedures (e.g., wearing masks, prohibitions on in-person practice or research). To provide Rorschach users with strategies to continue using it during the pandemic, the R-PAS authors developed two sets of guidelines (available at www.r-pas.org). The first consists of slightly modified administration guidelines for clinicians and researchers who were able to conduct in-person psychological testing with physical distancing. The second set of guidelines is for completing an assessment remotely using a videoconferencing platform. These guidelines require the respondent to have the inkblots in hand and to position themselves, potentially with a third camera, in such a way that the assessor can observe the respondent and the inkblots simultaneously. The guidelines discuss five possible scenarios. One involves just the respondent and the assessor; the others also involve a facilitator, who could be a member of the household or a quasi-professional aide to the assessor. Depending on circumstances, the facilitator could be in the room with the respondent during testing, which is less desirable, or on site and available if needed but not in the room. The assessor can implement these four options either in the respondent’s residence or at a clinical setting near the respondent. Each option has its own challenges and benefits, though they all encompass physical inkblots in the respondent’s hands and use of videoconferencing software to link the assessor with the respondent. Research has not explored whether these kinds of remote administration modify the assessment experience sufficiently to alter normative expectations. However, the R-PAS developers suggested that these designs sufficiently mirror in-person assessment to support their cautious use in practice.

A final option is more notably deviant from traditional in-person assessment with the respondent holding the inkblots in hand. This entails having a self-contained method for remote administration using inkblot stimuli presented to the respondent electronically on an appropriately sized device, while the assessor and the respondent link to each other via videoconferencing software. The R-PAS authors developed a new electronic app for this purpose. The app addresses several challenges. First, in partnership with Hogrefe, the publisher of the inkblots, it uses official electronic version of the original stimuli, in their precise color and shading. Second, it provides a screen calibration tool to ensure the inkblot images display at the correct size on the respondent’s device. Third, it protects intellectual property and test security by ensuring the images are not available in browser cache or via browser history and by ensuring they are inaccessible to either the respondent or the assessor once the session has been ended. Fourth, it provides options to the respondent for card turning. Fifth, the assessor controls movement from one card to another.

Finally, the remote administration app is an option within an electronic app that also is for use with traditional in-person assessment. The app provides a fully encrypted interface that links with user accounts on the R-PAS site, progresses through the phases of administration, allows the assessor to move around within or across phases, provides the option for speech to text transcription, and allows the assessor to add or delete responses and to score response behaviors related to card turning. It also provides image mark-up and annotation tools to complete an electronic version of a location chart and a place for the assessor to take notes on each response. Once the assessment is complete, the app securely sends all the responses information to the R-PAS site where it pre-fills the coding interface with relevant information, such as the structure of the responses, codes already assigned, the response and clarification phase communications, and, if present, notes from the assessor and card image annotations.

In the current study, we examine the remote use of the online app, given its relevance for potential use during the pandemic, as well as its remarkable flexibility by allowing the assessor and the respondent to reside anywhere rather than requiring them to be in the same room for the assessment. Since our sample was non-clinical, the procedure did not require help from facilitators.

### This Study

Although the remote administration app solves many technical hurdles, an important empirical question remains. Does the Rorschach administered remotely in a fully electronic format yield different information than when it is administered in-person with the cards in hand? Our pilot study begins to address this question by focusing on complexity, which is the “first factor” of all Rorschach scores and “the most important thing that makes one person look different from another person” on the Rorschach (Meyer et al., 2011, p. 319). In R-PAS, this variable is scored based on Viglione’s (1999) conceptualization of it as “the amount of productivity, precision, differentiation, and integration involved in the aggregate of all the responses” (p. 259). It also is the dimension of engagement observed by Meyer (1992) and historically by others, which he defined as “cognitive and emotional investment in the task as opposed to more simplistic or efficient responding” (p. 129).

Research supports the R-PAS approach to interpreting complexity scores. Mihura et al.’s (2013) meta-analytic review found that the Rorschach variables closely related to Complexity, i.e. those that assess cognitive synthesis, richness, or engagement, were among those with the strongest empirical support. Additionally, an eye-tracking study by Ales et al. (2019) found that complexity correlated at *r* = .53, *p* = .000002, with the number of fixations occurring during the response phase of administration. As this parameter (fixations number) is a well-established proxy marker of cognitive engagement in the eye-tracking literature (e.g., Chen & Proctor, 2017; Jarodzka et al., 2010; Laeng et al., 2011), Ales et al.’s study strongly corroborates the R-PAS approach to interpreting complexity scores. Also consistent with R-PAS interpretive guidelines, a recent fMRI study conducted by Vitolo et al. (2020) found that delivering more complex as opposed to simpler Rorschach responses showed increased activity in the dorsal attention network (*d* = .43, *p* < .01), a brain pathway deemed to be responsible for goal-directed or top-down attentional processes (Corbetta & Shulman, 2002; Ptak, 2012; Vossel et al., 2014).

Given that complexity is the Rorschach variable that defines the largest source of variability in the test, and considering its strong evidence base, we determined that it should be the primary focus of our first attempt to study whether administering the Rorschach remotely rather than in-person influences the respondent’s overall approach to the test. This study thus administered the Rorschach remotely to Italian adult community volunteers and evaluated the extent to which the complexity scores departed from R-PAS normative expectations derived from in-person administrations before COVID-19. Additionally, for exploratory purposes, we also examined the average scores of all other Rorschach variables included in the R-PAS profile pages of scoring output.

Ideally, to test possible differences between the remote vs. in-person administration formats, the R-PAS scores generated by our sample should be compared against those produced by an equivalent but independent in-person sample matched on key control variables (e.g., overall level of wellbeing). Alternatively, the same individuals could be administered the Rorschach twice, one time using the standard, in-person administration and one time using the remote administration format. However, when we designed and conducted this study, neither of these options was available because of COVID-19-related restrictions (lockdown, etc.). Accordingly, we opted to compare our newly collected data against those of R-PAS normative reference values, which—as noted above—were derived from in-person administrations before COVID-19. Although sub-optimal, this choice was justified by the fact that the vast majority of Rorschach variables is presumably influenced by stable personality traits rather than by short-lasting and unstable psychological states (Exner, 2003; Meyer et al., 2011; Sultan et al., 2006; Viglione & Hilsenroth, 2001). In fact, a meta-analytic study by Grønnerød (2003) found that on average Rorschach scores generate a test–retest stability coefficient of *r* = .64 over an interval of slightly more than 3 years. Along similar lines, a more recent study by Freitas and Pasian (2018) found that the scores generated by 88 volunteers over an interval of 15 years were similar across the two administrations. This was particularly true for variables more closely associated with the overall engagement with the task, which is the main focus of the current study. For instance, the number of responses correlated at *r* = .72, which was the highest correlation value among all presented results. Therefore, since the Rorschach appears to be related to stable personality traits rather than situational conditions and temporary affects, we believe that, in the lack of a sample collected with in-person administration during COVID-19 outbreak, the comparison with pre-pandemic R-PAS normative reference values may be a viable alternative.

As no published study had ever investigated the effects of administering the Rorschach remotely, we did not have any sound a priori hypotheses for this study. Thus, we speculated that individuals taking the Rorschach remotely would show a level of engagement—and thereby a complexity score—similar to that found with the standard in-person administration format, simply because we did not have any data suggesting otherwise. On the other hand, we also anticipated that R-PAS variables reflecting body-related preoccupations, social isolation, or reduced mental activity (e.g., anatomy-related content, passive movements) could perhaps slightly depart from normative expectations as a result of the ongoing pandemic.

## Method

### Participants

The sample consisted of 60 Italian young adults who were recruited across Italy. Table 1 provides basic demographic information for them and for the R-PAS international reference sample that established the normative expectations to which they are being compared. As the table suggests, our sample can be characterized as younger, more educated, generally single, and of one ethnic background relative to the norms, which are older, less educated, generally married, and multi-ethnic. In addition, unlike the R-PAS norms, about half of our sample (53.3%) were students. Nevertheless, it should be pointed out that empirical research shows that adulthood age, ethnicity, and gender have minimal to no influence on R-PAS scores (Meyer et al., 2014).

**Table 1:** Descriptive data in this study sample (*N* = 60) and in the reference sample (*N* = 640)

	**Study participants (** ***N*** ** = 60)**		**Reference sample (** ***N*** ** = 640)**	
**Variable**	**M**	**SD**	**M**	**SD**
Age	25.75	4.51	37.3	13.4
Years of Ed	16.05	2.03	13.3	3.6
	**Proportions**		**Proportions**	
Gender				
Male	43.3		44.7	
Female	56.7		55.3	
Ethnicity				
White	100		66.8	
Black	-		2.6	
Hispanic	-		8.7	
Asian	-		2.6	
Other or mixed	-		19.4	
Marital status				
Single	86.7		35.5	
With partner	10		1.6	
Married	1.7		52.3	
Separated	-		1.6	
Divorced	1.7		6.8	
Widowed	-		2.3	

As with the R-PAS norms, individuals in our sample may be characterized as “non-clinical,” as none had a history of psychological or psychiatric disorders with the exception of one person who reported a previous post-partum depression diagnosis. Inclusion and exclusion criteria required that participants (a) had never been administered the Rorschach and had no prior knowledge of the test; (b) were not regularly taking psychoactive or psychotropic drugs; (c) did not have dyschromatopsia, achromatopsia, and/or color blindness; and (d) had an electronic device (e.g., PC, laptop) large enough to see the Rorschach cards in the correct size dimension (i.e., 9.5 × 6.75 inches, or 24.13 × 17.145 cm). All of the above information was collected through a dedicated socio-demographic form that participants had to answer before taking part in the study. All participants used their devices and were in their own homes during Rorschach administration.

### Measures and Interrater Reliability

The R-PAS interpretive output reports scores from 60 Rorschach variables, located into two profile or summary pages. Variables with the strongest research and behavioral support are listed on page 1; those with less support or behavioral foundation are listed on page 2. The scoring program (www.r-pas.org) calculates all protocol level summary scores based on the codes entered at the response level (i.e., response by response) by the examiner; then it assigns percentiles based on normative reference data and converts those to a standard score (SS) metric (*M* = 100; *SD* = 15) for visual plotting. As such, the closer a score is to 100, the less it departs from normative expectations. The normative reference sample is heterogeneous in terms of culture and geography. Approximately one-fifth of the sample came from the USA, two-thirds from nine different European countries including Italy, and the remainder from Israel, Argentina, or Brazil. Thus, Western countries, cultures, and languages are well-represented within the sample.

To assess interrater reliability (IRR), 20 protocols were randomly selected and independently recoded by a rater who was blind to the original coding. The reliability of summary scores was quantified using an exact agreement intraclass correlation coefficient (ICC) for most variables. However, non-clinical samples generate very few codes representative of severe psychological problems (e.g., cognitive codes indicative of serious thinking problems; see Viglione et al., 2012), so we inspected the summary score distributions to identify those that had scores of just 0 or 1 (i.e., dichotomous variables) for either of the coders. For variables like these, there is enough information to assess whether the raters could code reliably the *absence* of that variable, but too few data points to test whether they could code reliably its *presence* (for more information, see Lewey et al. (2018)). Accordingly, for them, a contingency table was produced, and IRR was established via percentage of agreement and Gwet’s AC (Gwet, 2002), which is a variant of Cohen’s kappa.

The target variable of the current study, complexity, demonstrated an *excellent* IRR, with *ICC* = .95 (for common standards for characterizing *ICC* values, see Cicchetti, 1994). For the other 50 R-PAS variables whose distributions were non-dichotomous, *ICC* ranged from .47 to 1.00, with an average of .77 (*SD* = .14) and a median of .81. Specifically, 30 (60%) had *excellent* IRR (*ICC* > .74), 11 (22%) had *good* IRR (*ICC* > .60), and 9 (18%) had *fair* IRR (*ICC* > .40); none had *poor* IRR (*ICC* ≤ .40). For the remaining 9 dichotomous variables, percentage of agreement ranged from 75 to 100.0% (*M* = 89.2%; SD = 7.2%), and Gwet’s AC values ranged from .70 to 1.00 (*M* = .87; SD = .09). For these variables, one might say that our raters had a *good* to *excellent* agreement to code their absence, yet, there were too few scores in our data set to also test whether they would be able to reliably code their presence.

### Procedure

Prior to beginning recruitment and data collection, the bioethical committee of the University of Turin formally approved the research proposal. Prospective participants were recruited through word-of-mouth. They were first contacted (via email or phone) to make sure inclusion and exclusion criteria were met, and to obtain written consent^6^ and socio-demographic information. Next, the examiners—graduate students who had been trained by a member of the R-PAS Research and Development Group (last author) and supervised by an experienced R-PAS user (first author) in their data collection—scheduled an administration appointment.

Rorschach administration procedures closely resembled standard R-PAS guidelines, as much as possible given the online administration via the newly developed app. Once the video meeting with the respondent started through an online platform (e.g., WebEx, Zoom), the examiner shared a link to the remote card viewer with the respondent, and the respondent then shared their screen with the assessor. The assessor guided the participant through the test, as the card seen onscreen is determined by the assessor. The remote card viewer allowed both the assessor and the participant to see the cards and the respondent’s cursor on the card during clarification. In order for the respondent to view the cards at their true physical size, the participant was guided through a screen calibration process using a credit card, ID card, or piece of letter-size or A4 paper.^7^ R-PAS was administered in Italian since both the examiner and the examinee were native Italian speakers.

Consistent with standard R-PAS administration, each participant was asked to give two or maybe three responses per card. During the response phase (RP), the examiner encouraged a second response if only one was given and moved on to the next card if four responses were given, while providing a reminder of the desired number per card. On completion of the RP, the clarification phase (CP) was conducted as usual, with the participant seeing the cards again and helping the examiner to see what they saw during the RP. All protocols were typed as in-person and no examiner used the speech-to-text option that is also available on the app.

### Data Analysis

The primary aim of this study was to test whether the average complexity score significantly departed from SS = 100, which is the average score one should see if our data perfectly matched R-PAS normative expectations. Because null hypothesis significance testing cannot provide support for a true null hypothesis and can only prove it wrong when that is the case (Altman & Bland, 1995), we also implemented Bayesian statistics. Specifically, we used Rouder et al.’s (2009) JZS Bayes factor (JZS B) to estimate the relative posterior probability of the null and alternative hypotheses, given the data. We then interpreted this odds ratio based on Jeffreys’ (1961) criteria, which suggest that JZS B values of > 3, > 10, and > 30 should be characterized, respectively, as “some evidence,” “strong evidence,” and “very strong evidence” for the prevailing hypothesis.

Next, we examined—for exploratory purposes —the average scores of the other 59 variables on page 1 and page 2. To account for the multiple comparisons problem (Herzog et al., 2019), a Bonferroni-Holm correction was used for these one-sample *t*-test analyses. That is, we ordered our 59 results by their *p* value, and at the first step, we applied the “pure” Bonferroni correction (e.g., .05/59 = .000847 for α = .05) to the most statistically significant finding. We then sequentially adjusted the critical *p* value for the number of potentially true null hypotheses remaining in the set of analyses if the previous step was significant (for instance, if the first result survived the “pure” Bonferroni correction, then the second result was corrected for 58 potentially true nulls, e.g., .05/58 = .000862 for α = .05).

To characterize the effect sizes of the differences between our sample and the normative expectations, we used the standard deviation values of R-PAS norms to estimate our effect sizes and Glass’s delta to calculate *d*. Doing so provides a more accurate index of the extent to which remote administration departs from normative expectations than to use the standard deviation from both samples. When assessing normative equivalence for remote assessment, it is customary to consider differences of less than three tenths of a SD to be equivalent (e.g., Wright & Raiford, 2021), thus a *d* ≤ .30. It should be noted that these analyses were exploratory, as our study did not have the power to investigate such a large number of variables, given the relatively small sample size.^8^

For these exploratory analyses, the R-PAS interpretive output includes seven proportion scores, i.e., with scores other than the number of responses (R) as their denominator. For these scores, a value is computed only if there are at least three relevant codes, so that a respondent may produce a missing value on one or more of these scores. In this study, six of the seven proportion scores had a valid score on at least 40 of the 60 cases in our sample. However, the Mutuality of Autonomy Pathology Proportion (MAP/MAHP) only generated seven valid cases. For that specific proportion score, we substituted its numerator (i.e., Mutuality of Autonomy Pathology; MAP), so that all cases could be included in this analysis using the standard scores that allow comparison to the R-PAS reference norms.^9^

## Results

The average complexity score produced by our sample was SS = 100.83 (SD = 12.29; range = 73.00–130.00) and was not significantly different from the value of SS = 100.00 that one would see if our data perfectly matched R-PAS normative expectations, *t*(59) = .525, *p* = .601, *d* = .055. This result produced a JZS B value of 6.205, which indicates that the null hypothesis is greater than six times more likely than the alternative, given the data. Based on Jeffreys’ (1961) characterization of B, these data yield “some evidence” in support of the hypothesis that the R-PAS App to administer the Rorschach remotely produces the same complexity score, on average, as the in-person administration.

Results of the exploratory analyses of the other 59 scores are presented in Tables 2 and 3. Twenty-four variables produced statistically significant differences at a Bonferroni-Holm corrected alpha of .05. Effect sizes for these variables ranged from small (*d* = .29) to medium-large (*d* = .67). Thirty two variables were within the range that suggests equivalence (i.e., *d* ≤ .30). However, the average absolute value of *d* among these 59 variables was |.30| (*SD* = .20), and 23 variables had a significant difference and an effect size suggesting non-equivalence.

**Table 2:** One-sample *t*-test and effect size results for page 1 R-PAS variables under investigation

		***N***	***Min***	***Max***	***M***	***SD***	***t***	***df***	***p***	***d***
**Administration behaviors and observations**										
	Pr	60	89	119	96.9	9.0	− 2.64	59	.01	− 0.21
	Pu	60	96	138	103.2	11.1	2.21	59	.03	0.21
	CT	60	86	132	89.7	10.7	− 7.41	59	< .01**	− 0.68
**Engagement and cognitive processing**										
	R	60	73	130	104.7	8.5	4.29	59	< .01**	0.31
	F%	60	83	120	103.9	13.0	2.31	59	.02	0.26
	Blend	60	66	129	95.4	14.6	− 2.46	59	.02	− 0.31
	Sy	60	73	135	99.7	14.1	− 0.19	59	.85	− 0.02
	MC	60	64	130	100.4	13.4	0.23	59	.82	0.03
	MC—PPD	60	68	134	103.9	13.8	2.19	59	.03	0.26
	M	60	80	131	105.3	13.6	3.01	59	< .01	0.35
	M/MC	57	83	141	107.7	13.7	4.24	56	< .01**	0.51
	(CF + C)/SumC	40	84	135	93.9	13.6	− 2.87	39	.01	− 0.41
**Perception and thinking problems**										
	EII-3	60	75	126	106.9	14.2	3.76	59	< .01*	0.46
	TP-Comp	60	74	143	106.2	14.3	3.36	59	< .01*	0.41
	WSumCog	60	72	142	97.2	12.1	− 1.79	59	.08	− 0.19
	SevCog	60	79	135	95.6	5.3	− 6.46	59	< .01**	− 0.29
	FQ-%	60	94	113	110.1	14.9	5.27	59	< .01**	0.67
	WD-%	60	78	143	107.9	14.0	4.35	59	< .01**	0.52
	FQo%	60	82	143	90.6	12.2	− 5.96	59	< .01**	− 0.62
	Popular	60	66	111	95.1	10.6	− 3.58	59	< .01*	− 0.33
**Stress and distress**										
	YTVC'	60	73	119	97.4	13.3	-1.50	59	.14	− 0.17
	m	60	73	133	100.0	13.1	0.01	59	.99	0.00
	Y	60	84	131	98.0	12.9	-1.21	59	.23	− 0.13
	MOR	60	85	133	100.3	12.4	0.18	59	.86	0.02
	SC-Comp	60	86	123	93.4	12.9	− 3.96	59	< .01**	− 0.44
**Self and other representation**										
	ODL%	60	64	117	98.2	12.6	− 1.12	59	.27	− 0.12
	SR	60	74	124	96.9	11.1	− 2.16	59	.03	− 0.21
	MAP	60	87	127	98.9	11.0	− 0.75	59	.45	− 0.07
	PHR/GPHR	56	90	126	108.5	12.5	5.09	55	< .01**	0.57
	M-	60	75	136	106.7	13.3	3.89	59	< .01*	0.45
	AGC	60	95	143	100.3	14.7	0.17	59	.87	0.02
	H	60	74	136	102.4	14.1	1.30	59	.20	0.16
	COP	60	75	135	101.5	8.6	1.36	59	.18	0.10
	MAH	60	88	120	98.0	8.7	− 1.79	59	.08	− 0.13

^*^Statistically significant at .05 after applying Bonferroni-Holm correction

^**^Statistically significant at .01 after applying Bonferroni-Holm correction

**Table 3:** One-sample *t*-test and effect size results for page 2 R-PAS variables under investigation

		***N***	***Min***	***Max***	***M***	***SD***	***t***	***df***	***p***	***d***
**Engagement and cognitive processing**										
	W%	60	63	131	99.8	14.2	− 0.12	59	.91	− 0.01
	Dd%	60	75	122	97.4	12.1	− 1.66	59	.10	− 0.17
	SI	60	74	123	92.6	13.1	− 4.37	59	< .01**	− 0.49
	IntCont	60	81	112	91.1	9.9	− 6.96	59	< .01**	− 0.59
	Vg%	60	86	117	92.9	8.7	− 6.35	59	< .01**	− 0.48
	V	60	92	126	102.7	12.8	1.62	59	.11	0.18
	FD	60	88	122	93.2	8.8	− 5.98	59	< .01**	− 0.45
	R8910%	60	77	115	98.0	9.8	− 1.62	59	.11	− 0.14
	WSumC	60	70	116	93.8	12.9	− 3.72	59	< .01*	− 0.41
	C	60	95	114	97.5	6.5	− 2.93	59	< .01	− 0.16
	Mp/(Ma + Mp)	45	89	130	109.3	12.2	5.10	44	< .01**	0.62
**Perception and thinking problems**										
	FQu%	60	83	128	105.3	12.1	3.42	59	< .01*	0.36
**Stress and distress**										
	PPD	60	67	127	96.3	14.0	− 2.03	59	.05	− 0.24
	CBlend	60	91	126	97.4	10.2	− 2.00	59	.05	− 0.18
	C'	60	84	128	101.9	12.3	1.17	59	.24	0.12
	CritCont%	60	78	128	103.3	13.0	1.97	59	.05	0.22
**Self and other representation**										
	SumH	60	71	131	104.7	14.2	2.53	59	.01	0.31
	NPH/SumH	56	65	127	101.1	13.5	0.58	55	.56	0.07
	V-Comp	60	77	129	104.2	11.8	2.77	59	.01	0.28
	r (Reflections)	60	95	144	102.7	12.7	1.66	59	.10	0.18
	p/(a + p)	58	80	137	108.6	12.8	5.12	57	< .01**	0.57
	AGM	60	93	131	101.3	11.5	0.89	59	.38	0.09
	T	60	91	107	91.8	3.5	− 18.06	59	< .01**	− 0.55
	PER	60	92	109	93.1	4.3	− 12.44	59	< .01**	− 0.46
	An	60	85	136	109.7	13.8	5.46	59	< .01**	0.65

Vista (V) is also part of the stress and distress domain. However, we examined it just once in the engagement and cognitive processing domain

^*^Statistically significant at .05 after applying Bonferroni-Holm correction

^**^Statistically significant at .01 after applying Bonferroni-Holm correction

## Discussion

The ongoing COVID-19 pandemic has posed challenges to psychological and legal evaluations in applied settings. Recent studies have laid the groundwork in support of several psychological measures remotely administered (e.g., Brearly, 2017; Cullum et al., 2006; Galusha-Glasscock et al., 2016; Harrell et al., 2014; Parmanto et al., 2013; Smith et al., 2017; Wadsworth et al., 2018; Wright, 2018), identifying valid tests that could be used with a digital format (Corey & Ben-Porath, 2020; Wright, 2020; Wright & Raiford, 2021; Wright et al., 2020). Nevertheless, many clinicians, in transitioning from in-person to online practice, encountered difficulties, particularly for assessment using performance-based measures, as the presentation of online stimuli (e.g., Rorschach cards) poses additional challenges compared to self-report measures. These difficulties certainly contributed to mental health professionals being unsure of the feasibility of a tele-assessment and more reluctant to use it. On the other hand, psychological and legal evaluations could not stop, especially since so many individuals were likely experiencing some degree of distress due to isolation and the spread of COVID-19. In this respect, the state of emergency has served as a fuse to ignite applied interest in tele-assessment. However, no studies have assessed the lack of differences between standard vs. remote administration for performance-based personality measures.

Therefore, to update practitioners and researchers and inform them on how to use the Rorschach during the pandemic, the current article noted the guidelines developed by the R-PAS authors to administer the Rorschach in-person with physical distancing or remotely with the inkblots in hand. To extend those options further, this study pilot tested a newly developed app to conduct remote administrations using electronic inkblot stimuli developed by Hogrefe, the publisher of the original inkblots. Our findings may be summarized as follows: the general level of engagement shown by the test-takers when administered the Rorschach remotely with the new R-PAS app closely resembles that previously observed in the general population with “standard” in-person procedures. However, additional research is needed to appreciate the extent to which currently available R-PAS normative reference values are applicable to this new administration method.

Many studies have reported on the psychometric equivalence of administering tests via paper-and-pencil and computer formats using an in-person administration (e.g., Daniel & Wahlstrom, 2019; Daniel et al., 2014; Finger & Ones, 1999; Forbey & Ben-Porath, 2007; Menton et al., 2019; Pinsoneault, 1996; Roper et al., 1995). Fewer studies have focused on the comparison between in-person *vs* remote administration formats of psychological tests, particularly with respect to performance tasks (e.g., Brearly et al., 2017; Chuah et al., 2006; Marra et al., 2020; Wright, 2018). Because COVID-19 forced many practitioners to conduct their assessments remotely, we directed our research efforts to evaluate a newly developed R-PAS app aimed at allowing remote Rorschach administrations using electronic stimuli. This was done also because we believe that assessment at a distance will likely be a permanent part of psychological assessment even once the ongoing pandemic has subsided. In fact, it should be noted that this app could be used in the future also for in-person assessments, so that future studies could investigate its applicability also in the context of face-to-face administrations.

The most striking result of our study is that R-PAS variable Complexity, i.e., “the most important thing that makes one person look different from another person” on the Rorschach (Meyer et al., 2011; p. 319), generated a virtually identical average score, when compared to normative reference values generated via standard, face-to-face administration. Both the small effect size of this comparison (*d* = .055) and its relatively large Bayes factor value (JZS = 6.205) suggest that, overall, the Rorschach task should not change dramatically, when one takes it in-person at an office with the cards in hand versus electronically and remotely from home via video link. This finding is consistent with emerging research suggesting that other performance-based tests yielded similar results when administered in-person *vs* online and remotely (Brearly et al., 2017; Wright, 2018; Wright & Raiford, 2021). It is worth mentioning, however, that while previously published studies focused on tests investigating maximum performance, as far as we know, ours is the first to examine a typical performance measure.

Nevertheless, our study should not be taken as evidence that one can use the newly developed R-PAS remote app with no need to make any adjustments or refinements to existing R-PAS norms. Indeed, a first issue to keep in mind is that our comparison against R-PAS normative reference values is not optimal, as not only the administration format (in-person *vs* remote) but also the general context in which the data were collected (before *vs* during COVID-19) differ between the two data sets under examination. As such, even though Rorschach variables—especially those related to complexity—should not be dramatically affected by the different administration contexts, additional studies adopting a test–retest approach or random assignment to administration format are necessary before making any determination with regard to the suitability of extant Rorschach norms for the newly developed R-PAS remote app. The results of our pilot study should therefore be considered as preliminary and our conclusions as tentative. Additionally, our exploratory analyses inspecting all other scores from the R-PAS interpretive profile pages revealed that 23 variables generated a significant difference and an effect size suggesting non-equivalence (*d* > .30). Although we do not have any conclusive evidence to support our opinion, we believe that some of these discrepancies could be related to using the app, to testing being conducted in the comfort of one’s own home, or to the psychological consequences of the ongoing pandemic and related lifestyle changes.

For instance, relative to the R-PAS norms collected before COVID-19, our sample was more prone to concerns about their physical integrity (An), showed more idiosyncratic perceptions (FQ-%, WD-%, FQo%), were more cognitively ideational as opposed to reactive to bright and provocative stimuli (M/MC, WSumC), and generated more representations of passive activity (Mp/[Ma + Mp], p/[a + p]). These qualities could suggest that people in our sample were inside, wary of contact with others, ruminative rather than buoyant, and seeing their preoccupations in the cards rather than the conventional things people often notice during more normal times.

Other differences might reflect the modified format to present the stimuli remotely. This could explain, for example, why our sample was less likely to act on the perceptual environment by modifying the presented orientation of the inkblot stimuli (CT) and less prone to touch-related tactile representations (T). Third, testing completed while at home and at a distance from the assessor rather than in an office and adjacent to the assessor may contribute to a reduction in defensive assertions of personal knowledge (PER). All these considerations, however, are quite speculative at this time, given the small size of our sample. As such, additional research would be beneficial to clarify the extent to which these discrepancies from normative expectations really represent a true effect and the extent to which any true (i.e., replicating) effect is due to the mode of administration rather than the pandemic or the modified setting for the testing.

It is important to also underscore that even if non-clinical volunteers were to produce nearly identical R-PAS scores when administered the Rorschach in-person *vs* remotely with electronic stimuli, empirical evidence attesting to the validity of this remote administration format would still be needed. The generalizability of our findings to other cultural contexts also might be questioned, given that our pilot study only included a relatively small group of young and largely single Italian volunteers. As such, although R-PAS scores seem to be unaffected by the nationality and ethnicity of the test-taker or adult age (Meyer et al., 2015), additional research conducted remotely using this app in different cultural environments would be beneficial. Although adult age has not shown an association with R-PAS scores, our sample consisted of mostly students, and our average age (25.7, *SD* = 4.5) was much lower than in the R-PAS norms (37.3, *SD* = 13.4), which may play a role in our findings.

Furthermore, another aspect that our pilot investigation could not address is the extent to which individuals suffering from cognitive or psychiatric deficits or unfamiliar with computers or videoconferencing (e.g., elderly patients) could comply with remote administration requirements. To answer this and many other similar questions, more research is clearly needed.

Overall, the pandemic has boosted the growth of tele-assessment given the pressing need for professional services delivered remotely to the benefit of many people (Wosik et al., 2020). Being able to administer the Rorschach remotely holds the promise for similarly benefitting those in need of assessment during the pandemic. However, administering the Rorschach in videoconference assessment would be useful beyond this moment of health crisis to encompass other circumstances, such as assessing individuals with limited mobility, those whose travel would require more cost than benefit, inmates, or patients living in areas with few or no professional assessors.

The past year and a half has also seen courts shifting to forensic mental tele-health assessment (FMTA; Drogin, 2020). Pandemic limitations have affected private clinics, hospitals, prisons, and the offices of attorneys and consultants (Wright et al., 2020), which have encouraged forensic evaluators to practice remotely (Levy, 2020). Drogin (2020) predict that FMTA will not disappear with the control of COVID-19, but rather will become increasingly prevalent in the years to come, as it offers many benefits, such as reduced travel expenses, more flexible scheduling, and service to rural or remote areas. Hence, it is crucial that psycho-legal evaluations adapt to the change that is occurring.

Rorschach assessment should be similarly adaptable. Although potential limitations need to be considered, such as access to the technology needed for administration (e.g., laptop), high-speed internet, cultural and personal considerations (e.g., familiarity with the technology), and the environment in which the examinee is located during administration, developing an online method for administering the Rorschach is essential as assessment becomes increasingly oriented towards an “online” methodology. In light of this, our study represents the beginning of a systematic effort to demonstrate that the Rorschach can be administered online. Although our preliminary results are far from conclusive, they appear to be promising in addressing the important question, “Can the Rorschach yield interpretively useful information, when administered remotely?”.

## Footnotes

1 The term *tele-health* refers to physical and mental health assessment, prevention, and intervention services provided remotely. Thus, tele-health refers to a broader spectrum than *tele-assessment*, which is defined as the practice of administering via tele-communication psychological measures traditionally administered face-to-face—or side-by-side in the same room (Krach et al., 2020).

2 On October 11, 2021, a PsycINFO (EBSCO) search limited to the last decade (2010–2021) for articles using the cue words “Rorschach” or “Rorschach test” found 1424 results.

3 Maximum performance measures provide a clear and correct solution, guidance for successfully achieving that end, limited response options, and instructional sets that motivate behavior, whereas typical performance measures provide a context for engagement with a task, but the task lacks clear standards for success, provides many response options, and leaves it to the respondent to determine the manner of engaging the task.

4 FSN = fail-safe *N*, which is the number of effect sizes with null results required to bring the observed significance of the summary effect size to a level above *p* = .05.

5 Between 40 and 48% of assessors use the Rorschach in their daily practice (Ackerman & Ackerman, 1997; Quinnell & Bow, 2001).

6 Participants were asked to carefully read an information sheet regarding the research they were about to participate and, if they agreed, to sign the informed consent form.

7 Participants select one of these commonly available objects of standard size and place the object against the screen to align it with a representative image of that object displayed on the screen. The subject adjusts the size of the object on screen to match the size of the physical object by moving a slide bar.

8 This study was designed to investigate complexity. We considered that with a power of .80, a *small* to *medium* effect size of *d* = .35, and an alpha value set to .05, a sample size of about 60 to 70 participants was needed to compute a one-sample *t*-test (Cohen, 1988). Wanting to test a notably larger number of variables, however, a larger sample size would be necessary in order to account for the multiple comparisons problem.

9 Previous studies have followed the R-PAS recommendation to use difference scores as substitutes for research purposes (e.g., Schneider et al., 2020). We did not because standard scores are not provided for them.
